# From Rice Husk Ash to Silica-Supported Carbon Nanomaterials: Characterization and Analytical Application for Pre-Concentration of Steroid Hormones from Environmental Waters

**DOI:** 10.3390/molecules28020745

**Published:** 2023-01-11

**Authors:** Petra Bianchini, Francesca Merlo, Federica Maraschi, Rosaria Brescia, Mirko Prato, Antonella Profumo, Andrea Speltini

**Affiliations:** 1 Department of Chemistry, University of Pavia, Via Taramelli 12, 27100 Pavia, Italy; 2 Electron Microscopy Facility, Istituto Italiano di Tecnologia, Via Morego 30, 16163 Genova, Italy; 3 Materials Characterization Facility, Istituto Italiano di Tecnologia, Via Morego 30, 16163 Genova, Italy

**Keywords:** rice husk ash, biomass, carbon nanomaterials, extraction, emerging contaminants, circular economy

## Abstract

Rice husk (RH) in the rice industry is often air-burnt to obtain energy in the form of heat and RH ash (RHA) residue. In this work, RHA was applied as a starting material to obtain silica-supported carbon nanomaterials, resulting in a new reuse of a globally produced industrial waste product, in a circular economy approach. The preparation involves ultrasound-assisted one-pot oxidation with a sulfonitric mixture followed by wet oven treatment in a closed vessel. A study of oxidation times and RHA amount/acid volume ratio led to a solid material (nC-RHA@SiO_2_) and a solution containing silica-supported carbon quantum dots (CQD-RHA@SiO_2_). TEM analyses evidenced that nC-RHA@SiO_2_ consists of nanoparticle aggregates, while CQD-RHA@SiO_2_ are carbon-coated spherical silica nanoparticles. The presence of oxygenated carbon functional groups, highlighted by XPS analyses, makes these materials suitable for a wide range of analytical applications. As the main product, nC-RHA@SiO_2_ was tested for its affinity towards steroid hormones. Solid-phase extractions were carried out on environmental waters for the determination of target analytes at different concentrations (10, 50, and 200 ng L^−1^), achieving quantitative adsorption and recoveries (RSD < 20%, *n* = 3). The method was successfully employed for monitoring lake, river, and wastewater treatment plant water samples collected in Northern Italy.

## 1. Introduction

Rice is the second most produced cereal in the world, and its cultivation is the main activity and source of income for several countries in the world. At the beginning of the 1960s, its annual production was around 215 million tons; in 2020, its annual production was around 750 million tons [1]. After harvesting the rice, the first step in the production chain is milling. During this process, approximately 80% by weight is retained as rice, broken rice, and bran, while the remaining 20% is retained as rice husk (RH) [2]. RH is non-food biomass composed of 15–28% silica and 72–85% other organic components such as lignin, cellulose, and hemicellulose [3]. Its high silica content makes it difficult to compost or use as manure or animal feed due to its low nutritional value [2]. As a residue, RH in the rice industry is often air-burnt, obtaining, in line with the principles of circular economy, energy in the form of heat and RH ash (RHA), made of ca. 10% unburned carbon and around 90% amorphous silica [2]. Some other ways to reduce its accumulation are uses as a supplementary cementitious material, fuel in kilns to produce bricks and clay products or in rice mills to generate steam for the parboiling process, additive in fertilizers, flooring, and the production of biochar [2,4]. On the other hand, RH has great potential as a low-cost precursor to produce valuable materials, and its advantages are sustainability and environmental friendliness, despite the high diversity of the agricultural RH around the world leading to a certain variation in its properties and composition [5]. RH proves to be a good precursor for other sorbent materials as it has a granular structure, insolubility in water, chemical stability, and high mechanical strength. Adsorption is favored both by its irregular surface, which makes adsorption possible in different parts of the material, and by the presence of carboxylic functional groups and silanols that promote bonding and give considerable cation exchange capacity. It also has various cavities of different sizes, which are evidence of the porous structure and the large specific internal area that further facilitates analyte adsorption [6,7].

In the last few years, environmental pollution and the need to conserve energy and resources have led to the search for new, inexpensive, and available multipurpose sorbent materials that can be found in agro-food waste and by-products as primary sources [6]. Urbanization, industrialization, and population growth lead to the release of various types of pollutants from different sources into the environment. Natural activity contributes in small part to water pollution, but basically, the main sources are anthropogenic sources such as untreated municipal wastes and effluents, agricultural releases, and industrial chemical wastes [8]. Besides the conventional water pollutants, emerging contaminants encompass a very heterogeneous group of natural and synthetic compounds, such as pesticides, antimicrobials, steroid hormones (SHs), personal care products, and a variety of pharmacologically active drug residuals. They are defined as natural and synthetic compounds, generally detected at concentrations of nanograms per liter to micrograms per liter in water, and their presence in aqueous bodies is of particular concern because their behavior and toxicity are still largely unknown and unregulated [9]. 

The aim of this work is to develop a carbon nanomaterial endowed with sorption affinity for organic compounds that can be present in environmental waters as emerging contaminants. For the preparation of the sorbent, RHA provided by a local company has been chosen as a raw precursor to directly obtain silica-supported carbon nanomaterials, thus also allowing a different reuse of an industrial by-product. Silica-supported C-based materials prepared via grafting onto SiO_2_ substrates, including polymers [10] and different carbon nanomaterials [11,12,13], are currently used for the extraction of contaminants from environmental, food, and biological matrices. 

In this work, after evaluation of the main operational parameters affecting the ultrasound-assisted oxidation of RHA, the primary solid product obtained, named nC-RHA@SiO_2_, was evaluated as solid-phase extraction (SPE) sorbent for the multiclass pre-concentration of steroid hormones (SHs) in environmental waters before HPLC-MS/MS quantification. Specifically, the SPE protocol was developed for the simultaneous extraction of estrogens, namely 17β-estradiol (E2), 17α-ethynylestradiol (EE2), and estrone (E1); progestins, namely progesterone (PROG), medroxyprogesterone acetate (M-PROG), and hydroxyprogesterone (H-PROG); androgens, namely testosterone (TST), and epitestosterone (Epi-TST); and glucocorticoids, namely CORT, hydrocortisone (H-CORT), prednisolone (PREDLO), prednisone (PRED), dexamethasone (DEXA), betamethasone (BETA), fluocinolone (FLUO), and triamcinolone (TRIAM). The method was applied to steroid residue determination in lake, river, and wastewater treatment plant (WWTP) effluent samples.

Besides nC-RHA@SiO_2_, the procedure leads also to a solution containing silica-supported carbon quantum dots (CQD-RHA@SiO_2_), which were characterized (morphology, particle size, chemical composition) for future application. 

## 2. Results and Discussion

### 2.1. Evaluation of Operational Factors for RH and RHA Conversion into Carbon Nanomaterials

The first part of this study was aimed at developing a preparation procedure for the synthesis of silica-supported carbon nanomaterials starting from RH, as described by Wang et al. [3].

In detail, 1 g of RH was pyrolyzed in a tubular furnace under nitrogen flow by following these conditions: heating ramp of 10 °C min^−1^ to 700 °C, isothermal condition for two hours, and cooling ramp of 10 °C min^−1^ to room temperature. 

After verifying that pyrolyzed RH did not show any adsorption capability towards the target SHs, the char (1 g) was subjected to oxidation [3] by ultrasound-assisted one-pot treatment in 30 mL of a sulfonitric mixture (H_2_SO_4_:HNO_3_ 1:2, *v/v*) [14], and different oxidation times were investigated, specifically 2, 4, and 8 h. Then, 450 mg of the oxidized char were re-dispersed in 65 mL of tridistilled water and submitted to a thermal treatment in an oven at 200 °C for 10 h inside a 100 mL Teflon-lined autoclave (see details in Section 3.2). The solid materials obtained by filtration (0.22 µm) were investigated in terms of morphology by TEM analysis, highlighting the presence of micrometric aggregates of spherical nanoparticles with a diameter between 20 and 40 nm for all materials (Figure S1, Supplementary Materials). To evaluate their adsorption capacity and extraction efficiency towards SHs, preconcentration tests were carried out in tap water enriched with the target analytes. In detail, 25 mg of each material packed in a 1 mL SPE column was conditioned with 5 mL MeOH, 3 mL tridistilled water, and 2 mL tap water, and then 50 mL of tap water spiked with 2 μg L^−1^ of each steroid was passed through. Elution was carried out with 2 × 1 mL MeOH (5% *v/v* FA). Quantitative analysis was performed using HPLC-MS/MS in multiple reaction monitoring (MRM) mode with quantification by standard additions on each SPE eluate. These experiments showed that most analytes were quantitatively eluted from the materials prepared at any oxidation times, but the percentage of the adsorbed analytes increased as oxidation times increased (Table 1); thus, the 8 h oxidative treatment was selected.

Moreover, SPE control tests were also carried out on RH char after oxidation without the thermal treatment in a closed vessel; these tests resulted in non-quantitative adsorption that proved the effectiveness of the thermal treatment.

These encouraging results led to the possibility of directly employing RHA—the bottom ash from RH combustion that is conveniently practiced by the rice industry to reduce waste and to obtain energy—as starting material for the preparation of the sorbent phase. Synthesis was carried out as described above, resulting in a solid material (nC-RHA@SiO_2_) as the major product and a filtered solution containing CQDs grafted to silica nanoparticles (CQD-RHA@SiO_2_, fully characterized in Section 2.2). 

The TEM analyses on nC-RHA@SiO_2_ showed dimensions similar to those of the material obtained from pyrolyzed RH, and the pre-concentration tests (25 mg of sorbent material, 50 mL tap water enriched with 2 μg L^−1^) highlighted quantitative adsorption and recoveries (65–110%, *n* = 3). Given these satisfactory results, it was decided to further study RHA, as a major goal of this work. The amount of ash in the oxidation process, i.e., the liquid-to-solid phase ratio, was evaluated to reduce the acid volume per gram RHA. In detail 1, 3, and 5 g of char were oxidized with 30 mL of sulfonitric mixture for 8 h. Full adsorption and quantitative recoveries (60–110%, *n* = 3) were observed for all three materials obtained, and hence the liquid-to-solid phase ratio 30 mL:5 g RHA was chosen. This is a remarkable point that makes the original preparation method by Wang et al. [3] quicker and more sustainable and at the same time allows a scale-up of the synthesis, with an RHA-to-product conversion yield of 70%.

The batch-to-batch reproducibility was assessed by recovery tests on eight batches independently synthesized according to the final procedure (see Section 2.2); recovery was quantitative for all drugs, and the RSDs did not exceed 15%, thus attesting a satisfying reproducibility of sorbent preparation.

Driven by these appealing results, nC-RHA@SiO_2_ was deeply characterized (Section 2.2) and applied as an SPE sorbent in environmental waters (Section 2.3 and Section 2.5).

### 2.2. Characterization of nC-RHA@SiO_2_ and CQD-RHA@SiO_2_

The BET analysis of nC-RHA@SiO_2_ showed a surface area of 16 m^2^ g^−1^. The TGA analysis (Figure S2, Supplementary Materials) shows that from 100 °C to 1000 °C, the material is stable with a weight loss of about 2% around 500 °C, in accordance with the small amount of carbon in nC-RHA@SiO_2_ evidenced by XPS analysis, reported in the following.

The zero-charge point (PZC) of nC-RHA@SiO_2_ was determined using the immersion technique [15], and the PZC value was observed at ~pH = 7.

**Figure 1:**
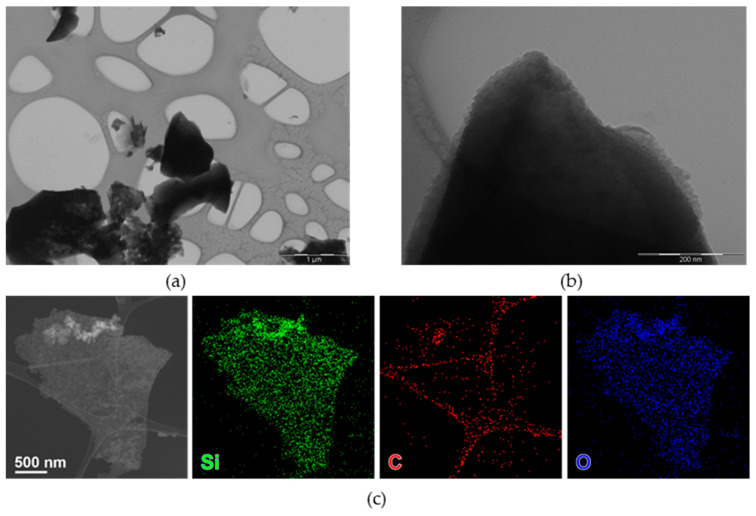
Bright-field TEM (BF-TEM) images of nC-RHA@SiO_2_ at different magnifications: (**a**) 25,000 magnification and (**b**) 200,000 magnification. HAADF-STEM images (**c**) of an aggregate of nanoparticles of nC-RHA@SiO_2_, partly suspended on a hole in the carbon support film, and corresponding STEM-EDS maps for Si (green), O (blue), and C (red).

nC-RHA@SiO_2_ was investigated by TEM-based techniques, showing few nanometer-size nanoparticles, often forming aggregates (Figure 1a,b). Compositional analysis (Figure 1c) shows the co-localization of Si, O, and C. 

**Figure 2:**
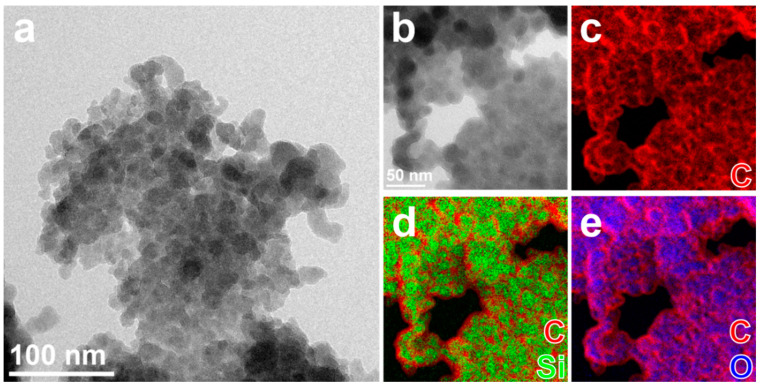
(**a**,**b**) Elastically filtered BF-TEM images of aggregates of nanoparticles of CQD-RHA@SiO_2_, partly suspended on holes in the carbon support film, and (**b**) zoomed-in area and (**c**–**e**) combinations of EF-TEM compositional maps for Si (green), O (blue), and C (red) acquired in the same aggregate.

A similar elemental distribution is observed for CDQ-RHA@SiO_2_, where the relative amount of C is increased compared to the sample nC-RHA@SiO_2_ (Figure S3 and Table S1, Supplementary Materials), and a closer look shows that each of the particles consists of a core of oxidized silicon (≈10 nm diameter) coated by a thin (≈3 nm thick) carbonaceous layer (Figure 2). High-resolution TEM analysis (not reported here) shows that the particles are amorphous.

**Figure 3:**
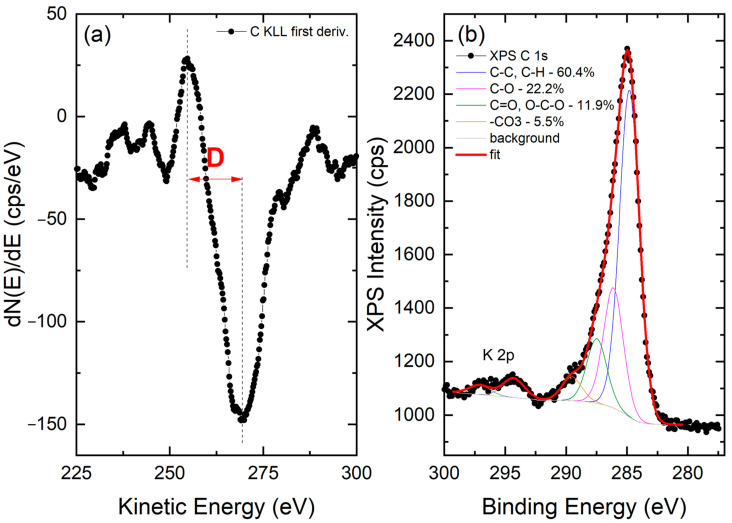
(**a**) First derivative of the C KLL X-ray-induced Auger spectrum acquired on the nC-RHA@SiO_2_ sample. The positions of the most positive maximum and of the most negative minimum are marked as vertical dashed lines. The distance in energy between the two positions defines the so-called D parameter, as marked. (**b**) XPS C 1s spectrum collected on the same sample. The results of the best fitting procedure are reported as well.

XPS analysis was carried out to shed light on the chemical state of the carbon fraction of nC-RHA@SiO_2_. Analysis of the survey scan (Figure S4, Supplementary Materials) suggests that carbon represents approximately 10 at.% of the surface composition of the nC-RHA@SiO_2_. The remaining 90 at.% is attributed to Si (approximately 27 at.%) and O (approximately 59 at.%), as well as traces of other metals (mainly Mg, Ca, Fe, and Mn). To infer the hybridization state of carbon, the X-ray-excited C KLL Auger spectrum was acquired and analyzed. As reported in the literature, the analysis of this spectrum is indeed more diagnostic of the carbon sp^2^ and sp^3^ configuration than the XPS C 1s spectrum, in which very similar position values are expected for the different hybridization of C [16]. The so-called D-parameter, defined as the distance in energy between the most positive maximum and most negative minimum of the first derivative of the C KLL spectrum, was therefore estimated in the case of nC-RHA@SiO_2_, resulting in a value of approx. 14.5 eV, indicative of an sp^2^ content in the 12–15% range [16]. Moreover, by analyzing the XPS C 1s spectrum, we were able to identify the presence of oxygenated carbon functionalities. As shown in Figure 3, besides the main component centred at 284.8 ± 0.2 eV and ascribable to C-C and C-H bonds, three other C species are present. These are ascribable to C-O (peak at 286.1 ± 0.2 eV), C=O/O-C-O (peak at 287.5 ± 0.2 eV), and -CO_3_ (peak at 287.5 ± 0.2 eV) groups [17].

Similar XPS analyses were also conducted on CQD-RHA@SiO_2_. In this case, the sample is characterized by a higher carbon content (around 18 at.% of the surface composition), but also by a relatively high K content. The presence of K effectively precludes the possibility of analyzing the C KLL Auger spectrum, because of the overlapping with the K LMM Auger peak. Analysis of the XPS C 1s spectrum (not reported) revealed the presence of the same oxygenated carbon functionalities as seen in the nC-RHA@SiO_2_ case, even if in different relative amounts (C-C/C-H: 53.5%; C-O: 11.5%; C=O/O-C-O: 13.6%; -CO_3_: 21.4%).

**Figure 4:**
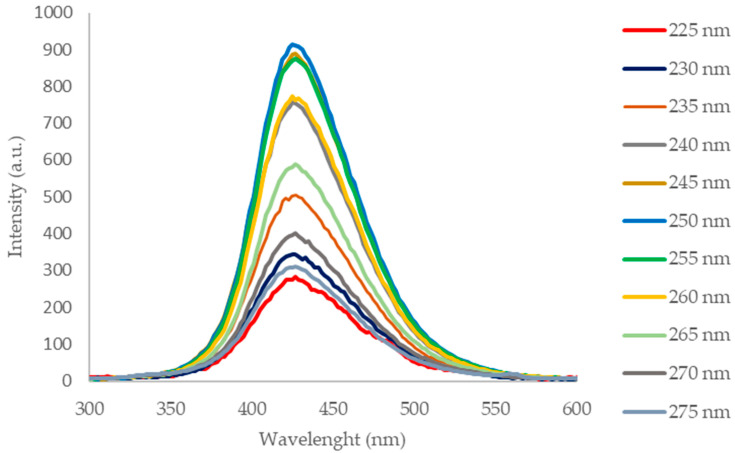
Photoluminescence spectra of CQD-RHA@SiO_2_ in the λ_ex_ 225–275 nm range.

The photoluminescence spectra (Figure 4) of the CQD-RHA@SiO_2_ solution acquired in the range λ_ex_ 225–275 nm show a maximum emission at 425 nm with a single symmetrical band that is evidence of homogeneity of the suspended nanoparticles. The optical features in terms of incident radiation absorption and emission in the visible range are in agreement with those of CQDs prepared by valuable precursors [18,19].

### 2.3. Analytical Application of nC-RHA@SiO_2_ as SPE Sorbent in Environmental Waters

As described in Section 2.1, the explorative SPE experiments highlighted that adsorption and recoveries were quantitative on nC-RHA@SiO_2_, well supporting the use of an agro-food by-product as RHA (instead of RH char from pyrolysis) as the sorbent precursor.

After evaluating the key role of the carbon aggregates in terms of adsorption capability by control test on pure silica (Ads < 40%), SPE tests on nC-RHA@SiO_2_ were undertaken to evaluate the role of different parameters affecting the SPE process. Firstly, the sample volume was increased up to 100 mL, but semi-quantitative adsorption was observed using 25 mg sorbent. Working with 50 mg nC-RHA@SiO_2_, quantitative adsorption and recovery (R > 75%, RSD < 15%, *n* = 3) were achieved.

Elution, as another SPE key factor, was then investigated by evaluating the percent acidity of MeOH. For this, MeOH, MeOH (1% *v/v* FA), and MeOH (5% *v/v* FA) were tested (2 × 1 mL). Lower recoveries were obtained with pure MeOH compared to acidified MeOH, and no significant variations were found between the two concentrations of FA; thus, 1% FA was selected. The higher elution efficiency under acidic conditions compared to neat MeOH can be justified considering the sorbent PZC (~pH = 7), which resulted in a positively charged surface during acidic elution promoting the desorption of the SHs. Since these are neutral compounds, as it is well known, their affinity for the material is favoured when the sorbent surface is globally neutral (not charged), whereas it is reasonably weakened when the sorbent possesses a neat charge.

This outcome is also in agreement with the good adsorption observed in tap water at the native pH, which is around 7.5 with no need for pH adjustment.

Under neutral conditions, the sorption of SHs is driven by interaction with the carbon fraction of the sorbent, as experimentally evidenced by SPE control tests on bare silica (<40%). Besides apolar (hydrophobic) interactions, other interplays with the oxygenated functionalities of the carbon fraction, confirmed by XPS, and the analyte molecular structures cannot be excluded (Figure S4, Supplementary Materials).

Guided by these excellent results, the final procedure (50 mg sorbent, 100 mL sample, elution with 2 × 1 mL 1% *v/v* FA MeOH) was extended to different environmental waters such as lake, river, and UWWTP effluent waters. Recovery tests were carried out in triplicate at three concentration levels (200 ng L^−1^, 50 ng L^−1^, and 10 ng L^−1^), and unspiked samples were simultaneously analyzed, showing no peaks at the retention time of the target analytes. For the 10 ng L^−1^ and 50 ng L^−1^ concentrations, the eluates were evaporated (see Section 3.4). As shown in Table 2, satisfactory recoveries were obtained for all the different matrices also at the lowest concentration (10 ng L^−1^), with an overall enrichment factor up to 200, thus strengthening the applicability of the material for extraction of trace pollutants in real-contaminated waters. Good results are achieved by a small amount of sorbent, with operation in the micro-SPE mode [20] further reducing solvent use. 

**Figure 5:**
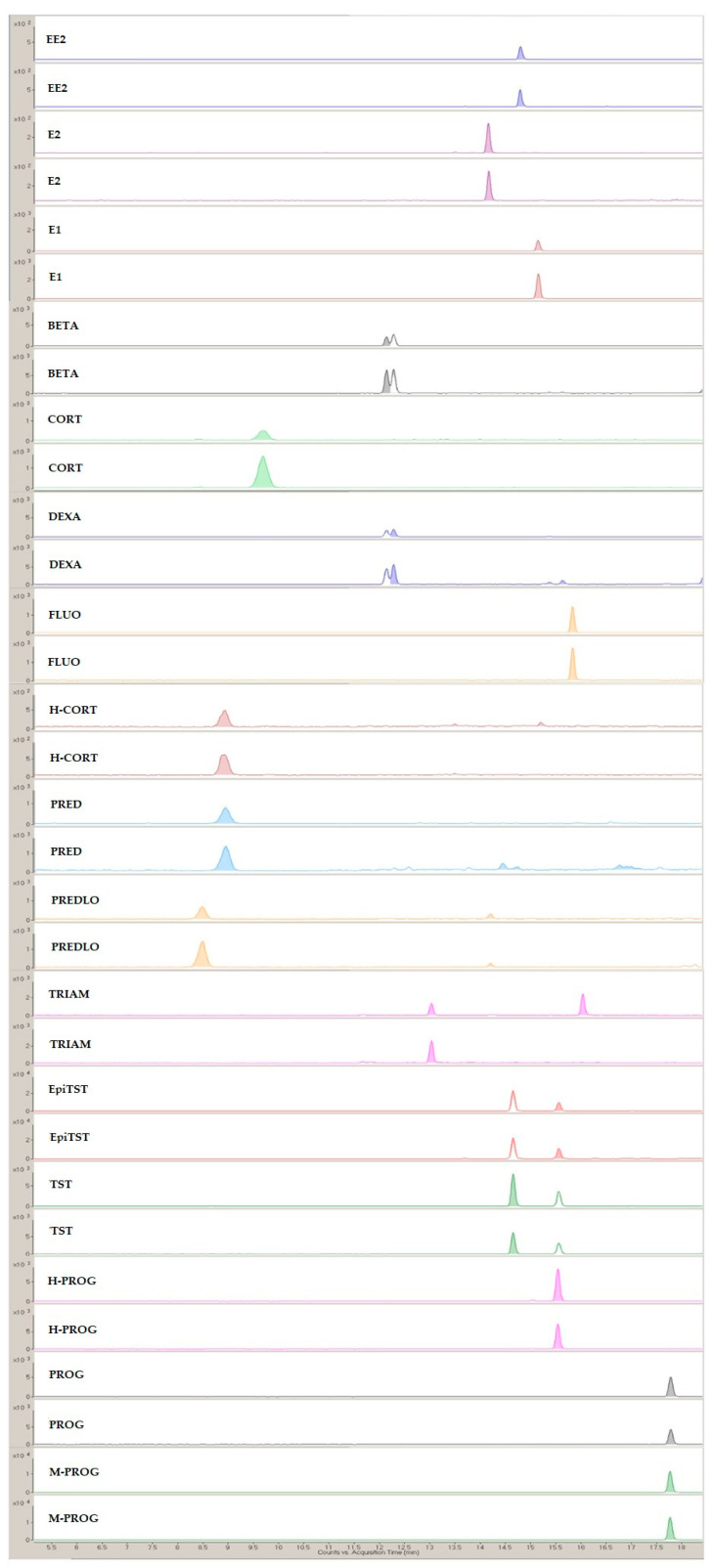
Representative MRM chromatogram from the analysis of the SPE lake eluates.

A representative MRM chromatogram from the analysis of the SPE lake eluates is presented in Figure 5.

Driven by these satisfactory results and the inherent characteristics of the developed sorbent, its applicability will be extended to the pre-concentration of other classes of emerging organic pollutants, e.g., fluoroquinolone antimicrobials. Additional work is in progress to enhance the sample throughput and to increase the sustainability of the procedure, further minimizing waste generation.

### 2.4. Analytical Evaluation of the Method

The entire analytical procedure was within-laboratory evaluated, according to the key features described in detail in Section 3.7.

Selectivity was guaranteed by MRM detection, which affords identification/quantification of the target compounds by using the most intense transition precursor/product ions of each compound (Table S2, Supplementary Materials). Furthermore, analysis of the eluates of the samples without spikes was carried out, showing that the target analytes of this method were not present. 

The matrix-matched calibration, for quantitation of the concentrations expected, was performed in the range 1–50 μg L^−1^ by five independent calibration curves in the eluate from blank environmental waters and provided good linearity (R^2^ > 0.9994 for lake sample, R^2^ > 0.9992 for river and UWWTP effluent samples).

Table 3 reports the method detection limits (MDLs) and method quantification limits (MQLs) obtained from each matrix-matched calibration curve for all the analytes, taking into consideration the whole sample treatment (see Section 3.7). Experimental MQL (10 ng L^−1^) was verified for all analytes in environmental waters.

The presence of matrix effect (ME) was assessed by comparing the slopes of matrix-matched calibration curves with those of the calibration lines obtained in the pure solvent, achieving the results reported in Table 3, and it was considered significative when not included within the method precision (RSD < 20%) [21]. 

### 2.5. Determination of SHs in Environmental Waters

The final procedure was used to investigate the presence of target SHs in environmental water samples, *viz*. from Garda Lake, Ticino River, and the wastewater effluent downstream the Vigevano urban wastewater treatment plant. Most analytes were detected (3–10 ng L^−1^) or quantified at concentrations in the range 10–23 ng L^−1^ (RSD < 20%, *n* = 3) (see Table 4). 

These results demonstrate that the proposed analytical method is a helpful tool for environmental monitoring, confirming that SHs are environmental water contaminants at concentration levels of tens of nanograms per liter, in agreement with our previous study [11]. 

## 3. Materials and Methods

### 3.1. Chemicals and Methods

All chemicals were reagent grade or higher in quality. Sodium hydroxide (>98%) and high-purity steroid hormone standards (E2, E1, EE2, TST, Epi-TST, PROG, M-PROG, CORT, H-CORT, PRED, PREDLO, BETA, DEXA) were supplied by Sigma-Aldrich (Milan, Italy). Analytical grade H-PROG was purchased from Steroids (Cologno Monzese, Italy), whereas TRIAM and FLUO were from Farmabios (Gropello Cairoli, Italy). SH molecular structures and Log*P* values are shown in Figure S5, Supplementary Materials. SH stock solutions of 1000 mg L^−1^ were prepared in MeOH and stored in the dark (4 °C), while working solutions (50–200 μg L^−1^) were prepared by dilution from a 2.5 mg L^−1^ solution, and they were renewed weekly. Ammonium fluoride (NH_4_F, for ACS analysis), anhydrous NaOH pellets (>97%), HPLC gradient grade methanol (MeOH), acetonitrile (ACN), and ultrapure water were purchased from Carlo Erba Reagents (Milan, Italy). Merck ultrapure acids (65% *w/w* HNO_3_, 96% *w/w* H_2_SO_4_) were used. Empty polypropylene tubes (1 mL) were from Merck (Milano, Italy). Ultrathin Films on Lacey Carbon were supplied by Nanovision (Brugherio, Italy). RH was supplied by a farm located in the Pavia province, and air-burnt RH (RHA) was supplied by an Italian rice company based in the Pavia province.

### 3.2. Materials Preparation

Five grams of rice husk ash (RHA) was sieved (100 mesh) and subjected to an oxidative treatment in an ultrasonic bath (Ultrasonic cleaner, 45 kHz, 80 W USC 200–2600, VWR International, Milan, Italy) with 30 mL of a sulfonitric mixture (H_2_SO_4_:HNO_3_, 1:2, *v/v*) for 8 h. The obtained solid char was vacuum filtered using a nylon filter (0.22 μm) and washed with plenty of tridistilled water (product yield 80% wt%). Four hundred fifty milligrams of the obtained material dispersed in 65 mL of tridistilled water was subjected to a thermal treatment in a high-pressure Teflon vessel (Acid Digestion Bomb with Teflon liner, bomb volume 23 mL, stainless steel body, Parr Instruments, Moline, IL, USA) at 200 °C (oven) for 10 h. The obtained suspension was then filtered through a 0.22 μm nylon filter and CQD-RHA@SiO_2_ were collected in the filtrate, while the final material used as sorbent, nC-RHA@SiO_2_, was dried at 60 °C for 2 h (yield 85% wt%). The overall yield of RHA conversion into nC-RHA@SiO_2_ was 70 wt%.

### 3.3. Silica-Supported Carbon Nanomaterial Characterization 

Specific surface area measurements on the samples were performed using a Sorptomatic 1990 instrument produced by Thermo Fisher Scientific. About 350 mg of powder was charged in the glass sample holder and degassed at 25 °C for 24 h. Subsequently, samples were cooled down at −196 °C and 2 adsorption–desorption cycles followed by a last adsorption run were performed (BET method, analyzing gas N_2_, 50 points for run; blank performed in He before the first adsorption run).

TGA measurements were performed with a TGA 1 Star System from Mettler Toledo (Novate Milanese, Italy). The heating rate was 10 °C min^−1^, and the range of temperature was from 25 °C to 1000 °C. Reactive gas was air (Sapio, Monza, Italy) at a flow rate of 100 mL min^−1^. For each experiment, about 10 mg of material was weighed and put into an alumina crucible. The TGA furnace was conditioned for 2 h to provide a suitable atmosphere for combustion.

Overview BF-TEM images were recorded on a Jeol JEM-1200 EX II instrument; a small amount of material (ca. 5 mg) was dispersed in water (ca. 1 mL) and sonicated for a few minutes, and a certain amount (ca. 5 µL) of the suspension was taken and deposited on the grid (400 mesh) through a micropipette.

Energy-filtered TEM (EF-TEM) and high-angle annular dark-field scanning TEM (HAADF-STEM) images and energy-dispersive X-ray spectroscopy data were acquired on an image-Cs-corrected Jeol JEM-2200FS S/TEM equipped with a Bruker X-Flash 5060 silicon drift detector-based EDS system.

The high silica content of CQD-RHA@SiO_2_ obtained from RH, evidenced by TEM analysis, was reduced by a NaOH treatment [3] and further characterized as follows: the CQD-RHA@SiO_2_ suspensions were drop-cast onto Cu grids coated with a holey carbon film. EDS mapping reported here was obtained by integration of intensities on the Kα peaks of C, O, and Si, and quantification was performed using the Cliff–Lorimer method and the same peaks. EF-TEM maps were obtained using the three-window method at the K core-loss edges of C and O, and the L_23_ edge of Si.

X-ray photoelectron spectroscopy (XPS) analyses were conducted with a Kratos Axis Ultra^DLD^ spectrometer, using a monochromatic Al Kα source operated at 15 kV and 20 mA. Survey scans were acquired with an analysis area of 300 × 700 microns and a pass energy of 160 eV. High-resolution analyses were carried out with the same analysis area and a pass energy of 20 eV. Spectra were charge-corrected to the main line of the carbon 1s spectrum set to 284.8 eV. Spectra were analyzed using CasaXPS software (version 2.3.24).

Emission spectra were recorded on a Cary Eclipse (Varian Ltd., Mulgrave, Victoria, Australia) spectrofluorimeter using a 1 cm × 1 cm quartz cuvette.

### 3.4. SPE Procedure

A multiposition vacuum manifold (Resprep manifold, Restek Corporation, Bellefonte, PA, USA) was used for the SPE. Fifty milligrams of nC-RHA@SiO_2_ were packed with 1 mL of MeOH in a 1 mL SPE column. The column, placed on the manifold, was conditioned with 5 mL of MeOH, 3 mL of tridistilled water, and 2 mL of tap water.

For recovery tests, 100 mL samples of environmental waters were spiked with each analyte to reach the final concentration (10 ng L^−1^, 50 ng L^−1^, 200 ng L^−1^). Elution was performed with 2 × 1 mL MeOH (1% *v/v* FA). For the 10 ng L^−1^ and 50 ng L^−1^ concentrations, the eluates were evaporated under air flow and resuspended with 0.5 mL of MeOH. The eluates were analyzed by HPLC-MS/MS (MRM mode).

### 3.5. Environmental Waters

Environmental waters were collected directly in amber glass bottles in the spring and summer of 2020 at 30–50 cm depth from Garda Lake, Ticino River, and UWWTP effluent in Vigevano (Pavia, Italy). Samples were stored in the dark (4 °C) and the physical–chemical parameters were measured for each sample, as reported in Table S3, Supplementary Materials.

### 3.6. HPLC-ESI-MS/MS

The HPLC apparatus Agilent 1260 Infinity coupled with an Agilent 6460C MS spectrometer ESI-MS/MS system (Cernusco sul Naviglio, Italy) was used for the analysis of the target analytes, according to previous papers [11,22]. Chromatographic separation was achieved with ZORBAX Eclipse Plus C18 Rapid Resolution (100 mm × 4.6 mm, 3.5 μm) preceded by a pre-column Supelco Supelguard Ascentis C18 (2 cm × 2.1 mm, 5 μm), both maintained at 25 °C (±0.8 °C). The sample injection volume was 10 μL, robotically injected by the HPLC autosampler. The flow rate was 0.5 mL min^−1^, with an analysis time equal to 20 min and a re-equilibration time of 7 min. Elution was performed with (A) aqueous 1 mM NH_4_F in H_2_O and (B) ACN, according to the following elution program: 30% B for 4 min, linear gradient to 85% B in 8 min, maintained for 5 min, and to 98% B in 0.5 min, maintained for 2 min. The MS operating parameters optimized by Agilent Mass Hunter Source Optimizer Software (Agilent, Santa Clara, CA, USA) were as follows: drying gas (N_2_) temperature 350 °C; drying gas flow 12 L min^−1^; nebulizer 50 psi; sheath gas temperature 400 °C; sheath gas flow 12 L min^−1^; capillary voltage 4000 V positive and 3000 V negative; nozzle voltage 0 V positive and 1500 V negative; electron multiplier voltage (EMV) 200 V positive and 0 V negative; cell accelerated voltage (CAV) 4 V positive and 1 V negative. Quantitative analysis was performed in multiple-reaction monitoring (MRM) mode, using the two most intense transitions of each compound (see Table S2, Supplementary Materials). All analytes were detected in the same chromatographic run by setting a polarity-switching tool. Typical MRM chromatograms of methanol standard solutions (10 μg L^−1^ of each analyte) are presented in Figure S6, Supplementary Materials. 

### 3.7. Analytical Evaluation of the Method

Selectivity was evaluated by the analysis of the blank environmental water with and without analyte spikes to evaluate the role of co-extracted components from the matrix.

To assess linearity in the matrix elutes, calibration curves were prepared for each analyte in pure solvent (MeOH) and in the elutes from each blank environmental water sample (matrix-matched calibration curves) [23,24] in the concentration range of 1–50 μg L^−1^. In ESI, the ionization efficiency of the analytes may be strongly altered by the co-eluting compounds, and different matrices can lead to different matrix effects; thus, ME was investigated in each extract from lake, river, and wastewater effluent samples, and it was calculated as follows:

(1) $$\mathrm{ME}\;\left(\%\right)=\left(\frac{b_{m}}{b_{s}}-1\right)\times \;100$$

where “b_m_” and “b_s_” are the slopes of the matrix-matched calibration curve and of the calibration line obtained in pure solvent, respectively, obtained by ordinary linear lowest squares regression (OLLSR). 

MDLs and MQLs (μg L^−1^) were calculated from the matrix-matched calibration curves as 3.3 and 10 times the ratio between the baseline noise away from the peak tail and the regression line slope, respectively, taking into consideration the whole sample treatment [11,22].

Independent recovery tests (*n* = 3) were performed on environmental water samples enriched with each analyte at three different concentration levels, namely low-quality control (LQC, 10 ng L^−1^), medium-quality control (MQC, 50 ng L^−1^), and high-quality control (HQC, 200 ng L^−1^), to evaluate trueness and precision. Trueness was calculated as the ratio of the concentration obtained after the entire procedure on the spiked sample and the concentration expected. The inter-day precision was expressed as RSD% associated with the mean recovery.

Robustness was evaluated by small and deliberate variations in operating conditions, whereas instrumental carry-over was checked by injections of SPE eluting solution (MeOH 1% *v/v* FA) and SPE eluates from each blank water sample as control blanks after consecutively analyzed samples [22]. 

## 4. Conclusions

RHA, obtained in the rice industry as a bottom ash of the RH combustion, has been successfully transformed into a working fixed-bed SPE sorbent (nC-RHA@SiO_2_) for the pre-concentration of emerging and hazardous pollutants, such as SHs. Indeed, the presence of carbon embedding oxygenated groups, shown by XPS and HAADF-STEM analyses, exerts a key role in the quantitative adsorption/desorption of the target SHs in natural waters. 

A simple and quick analytical method, based on a SPE procedure followed by HPLC-MS/MS quantification, was developed for the multiclass determination of SHs at environmentally relevant concentrations. 

Another important goal achieved in this work is the one-pot treatment involved in the RHA oxidation, with a remarkable reduction in acid consumption. Noteworthily, this procedure also led to added-value products such as CQD-RHA@SiO_2_ which will be tested in future work. 

## Figures and Tables

**Table 1:** Adsorbed percentage (% Ads) and mean recovery of analytes in the sample (% R, *n* = 2) observed testing the oxidized material at different times (2 h, 4 h, and 8 h) on 50 mL of tap water spiked with 2 μg L^−1^ of each analyte.

			Oxidation Time			
Analyte	2 h		4 h		8 h	
	% Ads	% R	% Ads	% R	% Ads	% R
EE2	70	69	67	51	99	98
E2	78	67	77	50	99	86
E1	78	64	85	55	99	82
BETA	53	47	66	61	93	88
CORT	28	25	71	52	98	94
DEXA	60	45	73	53	95	91
FLUO	48	46	53	46	99	99
H-CORT	11	9	55	9	99	97
PRED	51	35	70	50	80	78
PREDLO	34	27	61	52	99	96
TRIAM	35	27	55	27	96	92
EpiTST	70	70	78	55	99	95
TST	72	62	80	58	99	97
H-PROG	77	62	82	60	98	94
PROG	97	76	94	65	100	60
M-PROG	97	94	94	94	99	94

**Table 2:** Mean recovery values (% R, *n* = 3) with relative standard deviation (RSD%) observed in lake, river, and UWWTP effluent waters.

Mean Recovery (%) ^a^									
Analyte	Lake			River			UWWTP		
	200 ng L^−1^	50 ng L^−1^	10 ng L^−1^	200 ng L^−1^	50 ng L^−1^	10 ng L^−1^	200 ng L^−1^	50 ng L^−1^	10 ng L^−1^
EE2	93 (1)	104 (4)	83 (10)	81 (6)	101 (17)	99 (10)	117 (6)	80 (8)	78 (5)
E2	63 (11)	81 (9)	72 (15)	61 (9)	76 (14)	74 (14)	66 (14)	64 (13)	88 (12)
E1	63 (9)	65 (17)	100 (16)	57 (10)	69 (12)	119 (2)	76 (10)	71 (15)	104 (4)
BETA	97 (3)	107 (19)	104 (14)	84 (6)	99 (14)	113 (3)	112 (10)	75 (11)	97 (3)
CORT	79 (17)	83 (22)	101 (1)	59 (3)	55 (12)	68 (13)	106 (9)	64 (15)	65 (16)
DEXA	91 (12)	83 (15)	114 (10)	74 (7)	96 (14)	109 (3)	124 (1)	77 (14)	111 (5)
FLUO	97 (8)	106 (2)	72 (6)	91 (6)	108 (12)	122 (5)	112 (7)	83 (12)	120 (4)
H-CORT	84 (11)	95(7)	76 (10)	69 (7)	71 (8)	69 (15)	101 (11)	65 (8)	65 (8)
PRED	79 (16)	104 (6)	74 (10)	57 (6)	54 (15)	68 (11)	96 (7)	71 (12)	92 (2)
PREDLO	82 (10)	97 (20)	72 (5)	70 (4)	64 (18)	72 (11)	106 (9)	63(10)	84 (9)
TRIAM	91 (5)	100 (6)	66 (2)	93 (5)	83 (13)	102 (14)	119 (11)	76 (10)	107 (8)
EpiTST	94 (1)	109 (6)	87 (5)	94 (7)	92 (8)	87 (5)	105 (3)	70 (15)	86 (15)
TST	82 (13)	78 (20)	57 (11)	77 (8)	88 (4)	77 (18)	119 (12)	81 (13)	71 (10)
H-PROG	98 (10)	103 (2)	83 (9)	95 (1)	99 (7)	117 (9)	140 (9)	73 (13)	120 (4)
PROG	62 (6)	53 (19)	60 (10)	55 (20)	56 (12)	64 (6)	60 (12)	72 (20)	75 (7)
M-PROG	87 (11)	111 (6)	107 (15)	92 (16)	103 (7)	120 (6)	108 (4)	89 (11)	95 (12)

^a^ For 10 and 50 ng L^−1^ recovery tests, the SPE eluate was evaporated under air flow to dryness and reconstituted in 0.5 mL MeOH.

**Table 3:** ME (%) and calculated MDL and MQL values (ng L^−1^) from each matrix-matched calibration curve, taking into consideration the whole sample treatment.

Analyte	Lake			River			UWWTP Effluent		
	MDL	MQL	ME	MDL	MQL	ME	MDL	MQL	ME
EE2	0.05	0.1	<20	0.07	0.2	<20	0.01	0.03	−34
E2	0.04	0.1	<20	0.05	0.1	<20	0.01	0.04	−37
E1	2	5	36	0.1	0.3	<20	0.05	0.1	−32
BETA	0.03	0.1	<20	0.1	0.3	<20	0.003	0.01	−58
CORT	2	6	<20	3	10	−22	0.3	0.9	−46
DEXA	0.2	0.7	−37	0.6	2	<20	0.03	0.09	−46
FLUO	0.7	2	<20	1	4	26	0.06	0.2	−38
H-CORT	2	5	−28	3	10	−24	0.1	0.3	−39
PRED	0.1	0.3	<20	0.4	1	−27	0.06	0.2	−34
PREDLO	0.2	0.5	<20	0.2	0.8	−29	0.03	0.1	−66
TRIAM	0.2	0.6	<20	0.2	0.5	<20	0.02	0.06	−37
EpiTST	0.7	2	−37	0.8	2	<20	0.2	0.7	−44
TST	0.2	0.5	<20	0.09	0.3	<20	0.03	0.09	−63
H-PROG	0.8	2	<20	1	4	<20	0.02	0.07	−45
PROG	0.3	0.9	<20	0.6	2	−47	0.04	0.1	<20
M-PROG	1	3	−26	1	3	−47	0.08	0.3	23

**Table 4:** Nanograms per liter of SHs quantified in Garda Lake, Ticino River, and Vigevano UWWTP effluent water samples.

ng L^−1^ of SHs in Environmental Waters			
Analyte	Garda Lake	Ticino River	Vigevano UWWTP
EE2	<MQL	<MQL	<MQL
E2	<MQL	10	12
E1	12	12	17
BETA	<MQL	<MQL	<MQL
CORT	<MDL	<MDL	<MDL
DEXA	<MQL	<MQL	10
FLUO	22	<MQL	<MQL
H-CORT	12	<MQL	<MDL
PRED	<MQL	<MQL	<MQL
PREDLO	<MDL	<MDL	<MDL
TRIAM	<MQL	<MDL	<MQL
EpiTST	<MQL	<MQL	<MQL
TST	<MQL	<MQL	<MDL
H-PROG	17	17	14
PROG	11	10	13
M-PROG	23	16	13

## Data Availability

The data presented in this study are available on request from the corresponding author.

## Supplementary Materials

The following supporting information can be downloaded at: https://www.mdpi.com/article/10.3390/molecules28020745/s1, Figure S1: TEM images at different magnifications (25,000, 200,000, and 250,000) of the material after oven treatment from pyrolyzed RH oxidized at different times: (a) 2 h, (b) 4 h, and (c) 8 h; Figure S2: TGA profiles recorded on nC-RHA@SiO_2_; Figure S3: (a) HAADF-STEM image of a region in the sample CQD-RHA@SiO_2_, and corresponding STEM-EDS maps for Si (b), C (c) and O (d). For these regions, partly suspended on holes in the support carbon film, there is a clear co-localization of the three elements in the nanostructured material; Figure S4: XPS survey spectrum acquired on nC-RHA@SiO_2_; Figure S5: Molecular structures and Log*P* values of the target analytes; Figure S6: Typical MRM chromatogram of a SH standard solution (10 μg L^−1^ of each analyte) prepared in methanol.; Table S1: STEM-EDS compositional analysis of (a) nC-RHA@SiO_2_ and (b) CQD-RHA@SiO_2_ suspended on holes in the carbon support film; Table S2: MRM conditions for the HPLC-ESI-MS/MS analysis of SHs; Table S3: Physicochemical parameters of the water samples.

## Sample Availability

Samples of the compounds are not available from the authors.
