# Supplementary material for: From Rice Husk Ash to Silica-Supported Carbon Nanomaterials: Characterization and Analytical Application for Pre-Concentration of Steroid Hormones from Environmental Waters
Source: Molecules. 2023 Jan 11;28(2):745. doi: 10.3390/molecules28020745 (PMC9866712; doi:10.3390/molecules28020745)
Supplement: Supplementary file 1 [file molecules-28-00745-s001.zip › molecules-2116286-supplementary.pdf]

Supplementary Material

From Rice Husk Ash to Silica-supported Carbon Nanomaterials: Characterization and Analytical Application for Pre-concentration of Steroid Hormones from Environmental Waters

Petra Bianchini [1], Francesca Merlo[1], Federica Maraschi[1], Rosaria Brescia[2], Mirko Prato[3], Antonella Profumo[1], and Andrea Speltini[1*]

[1]*Department of Chemistry, University of Pavia, Via Taramelli 12, 27100 Pavia, Italy*
[2]*Electron Microscopy Facility, Istituto Italiano di Tecnologia, via Morego 30, 16163 Genova, Italy*
[3] *Materials Characterization Facility, Istituto Italiano di Tecnologia, via Morego 30, 16163 Genova, Italy*
[*]*Correspondence: andrea.speltini@unipv.it; 0382 987350*

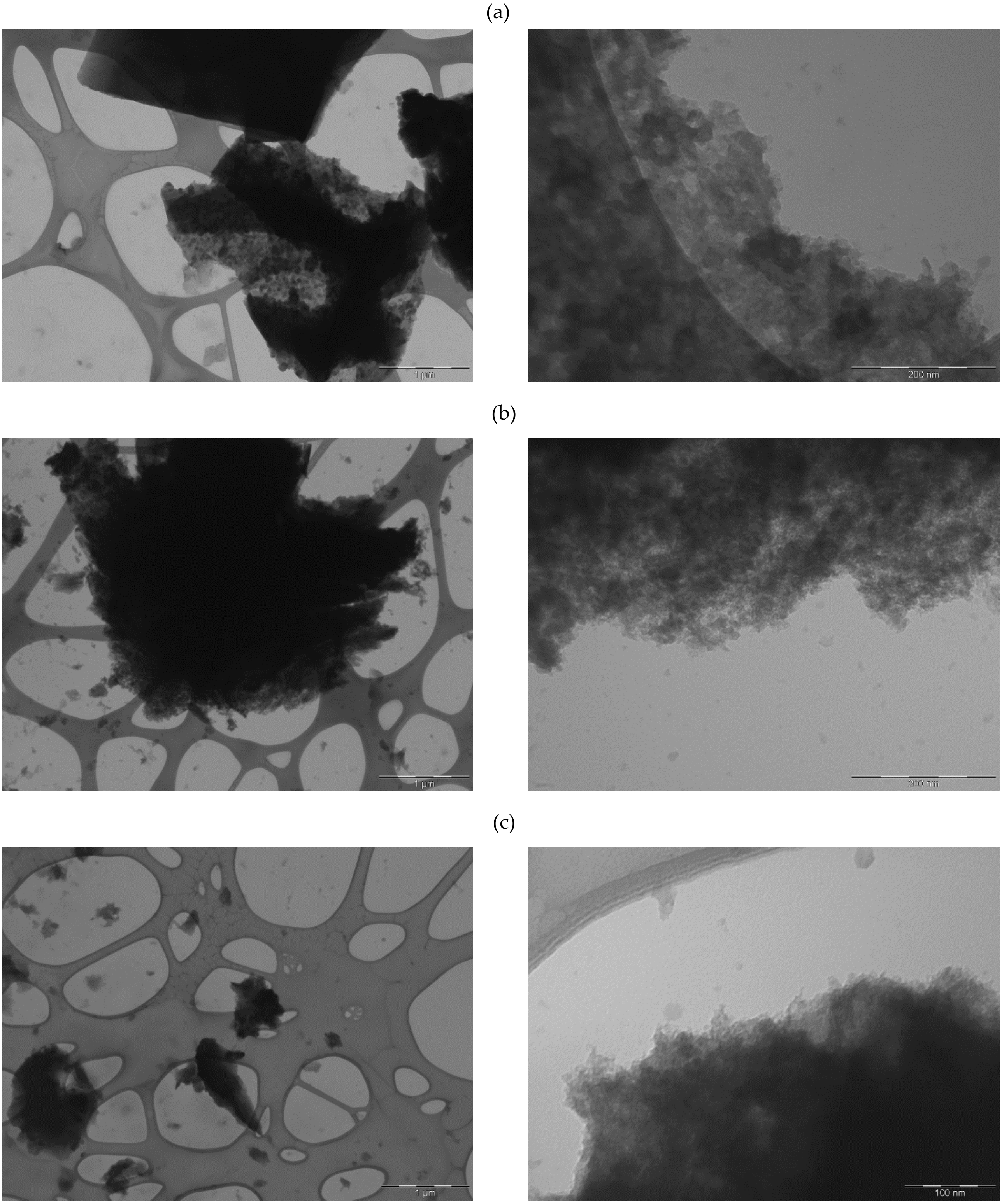

Figure S1. TEM images at different magnification (25,000, 200,000, and 250,000) of the material after oven treatment from pyrolyzed RH oxidized at different times: (a) 2 hours, (b) 4 hours, and (c) 8 hours.

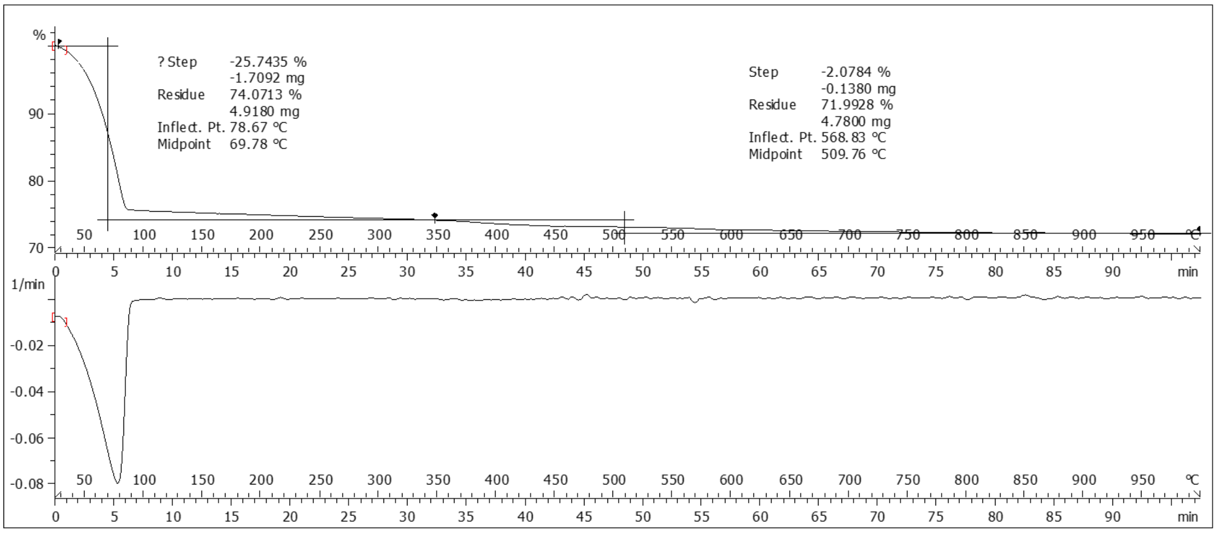

Figure S2. TGA profiles recorded on nC-RHA@SiO_2.

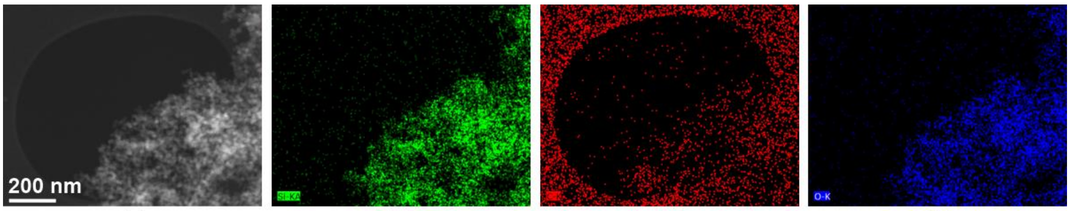

(a) (b) (c) (d)

Figure S3. (a) HAADF-STEM image of a region in the sample CQD-RHA@SiO_2, and corresponding STEM-EDS maps for Si (b), C (c) and O (d). For these regions, partly suspended on holes in the support carbon film, there is a clear co-localization of the three elements in the nanostructured material.

Table 1. STEM-EDS compositional analysis of (a) nC-RHA@SiO_2 and (b) CQD-RHA@SiO_2 suspended on holes in the carbon support film.

		**a**	
**Element**	**Series**	**Atom C. [at. %]**	**Rel. error (1 Sigma) %**
C	K series	23.08	20.80
O	K series	47.64	5.33
Si	K series	29.28	3.18
Cu	K series	0.00	
Ca	K series	0.00	
	Total	100.00	

b			
**Element**	**Series**	**Atom C. [at. %]**	**Rel. error (1 Sigma) %**
C	K series	49.64	5.77
O	K series	31.20	3.62
Si	K series	19.16	1.42
Cu	K series	0.00	
Ca	K series	0.00	
	Total	100.00	

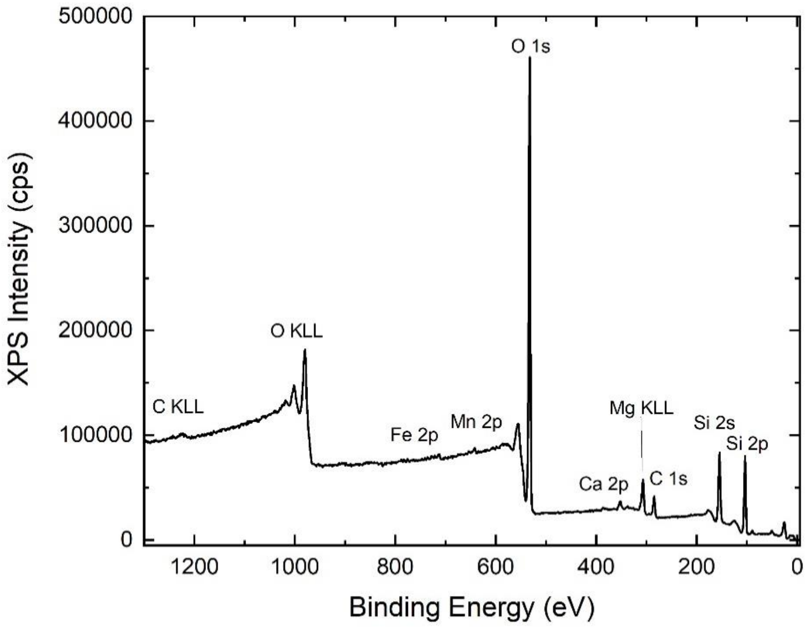

Figure S4. XPS survey spectrum acquired on nC-RHA@SiO_2.

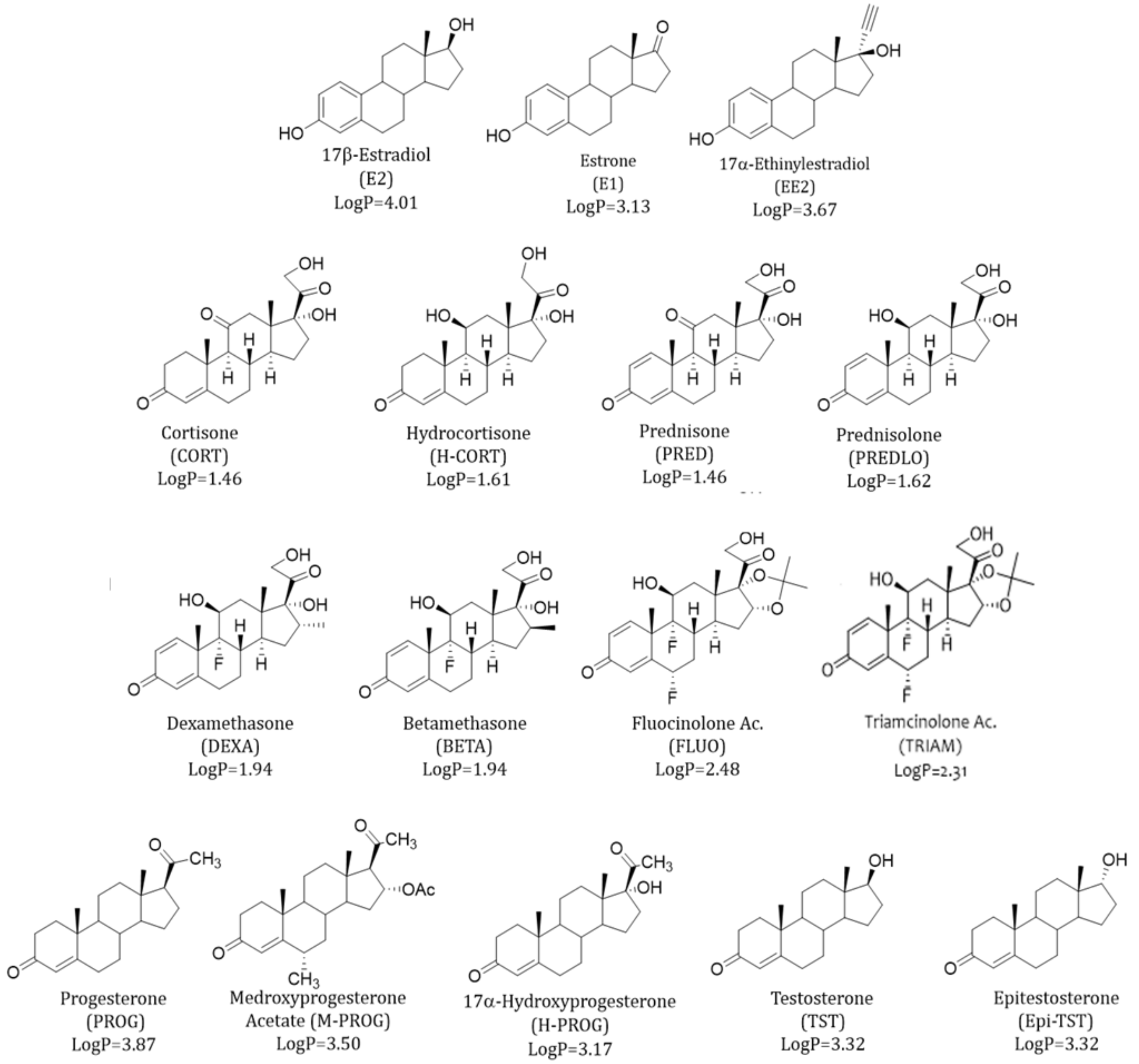

Figure S5. Molecular structures and Log*P* values of the target analytes.

Table S2. MRM conditions for the HPLC-ESI-MS/MS analysis of SHs.

Analyte	Precursor Ion (m/z)*	Product Ion (m/z)	Dwell Time (ms)	Fragmentor energy (V)	Collision energy (V)	Polarity
EE2	295	145.1	50	154	44	Negative
		143.1	50	154	68	
E2	271	183.3	100	166	50	Negative
		143.1	100	166	64	
E1	269	145.1	15	148	55	Negative
		143.1	15	148	60	
BETA	373	355.3	15	82	8	Positive
		147.1	15	82	24	
CORT	361	163.2	15	130	20	Positive
		105	15	130	48	
DEXA	373	355.4	15	76	4	Positive
		91	15	76	80	
FLUO	495	475.3	15	88	0	Positive
		337.3	15	88	8	
H-CORT	363	121	15	124	20	Positive
		91	15	124	68	
PRED	359	341.3	15	76	4	Positive
		147.1	15	76	24	
PREDLO	343	147.1	15	112	20	Positive
		91	15	112	72	
TRIAM	415	397.3	15	130	8	Positive
		91	15	130	80	
EpiTST	253	109	15	100	25	Positive
		97	15	100	20	
TST	289	109	15	76	24	Positive
		97.1	15	76	20	
H-PROG	331	109.1	15	118	28	Positive
		97.1	15	118	24	
PROG	315	109	15	94	24	Positive
		97	15	94	20	
M-PROG	387	327.4	15	106	8	Positive
		123.1	15	106	24	

*$[M–H]^-$ adduct for negative ion and $[M+H]^+$ adduct for positive ion.

Table S3. Physicochemical parameters of the water samples.

	Tap Water	Lake water	River water	UWWTP effluent	Garda Lake water	Ticino River water	Vigevano UWWTP effluent
pH	7.7	7.81	7.2	7.6	7.14	7.4	7.3
X 20 °C	270	59	344	164	278	178	294
HCO_3^-	-	-	176	70	-	86	65
Cl^-	5.0	2.2	11.3	5.5	13.3	5.3	31.0
NO_3^-	0.6	3.0	6.7	4.2	9.0	3.8	35.0
SO_4^{2-}	5	6.0	33.6	24.5	14.3	23.9	23.0
Ca	36.1	7.0	52.5	23.5	39	27.3	23.0
Mg	8.1	1.6	13.5	5.6	7.2	6.0	5.6
Sr	-	-	0.20	0.20	-	0.20	0.10
Na	12.5	2.7	10.0	5.7	9.5	5.1	26.5
K	-	-	2.6	1.4	-	1.4	5.3
SiO_2	-	-	15.5	2.0	-	3.6	15.0

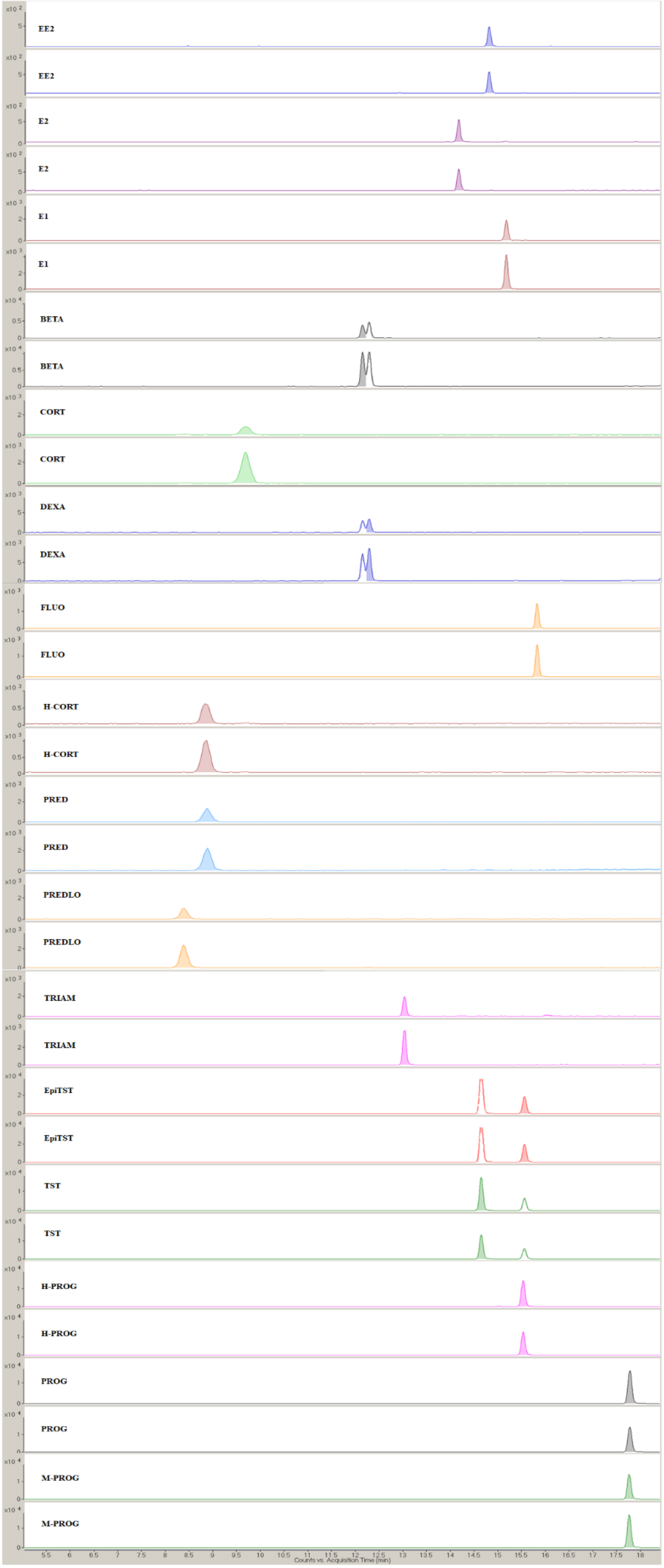

Figure S6. Typical MRM chromatogram of a SHs standard solution (10 µg L^{-1} of each analyte) prepared in methanol.
